# Reactions of *Plasmodium falciparum* Ferredoxin:NADP^+^ Oxidoreductase with Redox Cycling Xenobiotics: A Mechanistic Study

**DOI:** 10.3390/ijms21093234

**Published:** 2020-05-02

**Authors:** Mindaugas Lesanavičius, Alessandro Aliverti, Jonas Šarlauskas, Narimantas Čėnas

**Affiliations:** 1 Department of Xenobiotics Biochemistry, Institute of Biochemistry of Vilnius University, Saulėtekio 7, LT-10257 Vilnius, Lithuania; mindaugas.lesanavicius@gmc.vu.lt (M.L.); jonas.sarlauskas@bchi.vu.lt (J.Š.); 2 Department of Biosciences, Università degli Studi di Milano, via Celoria 26, I-20133 Milano, Italy; alessandro.aliverti@unimi.it

**Keywords:** ferredoxin:NADP^+^ oxidoreductase, *Plasmodium falciparum*, quinones, nitroaromatic compounds, aromatic *N*-oxides, oxidative stress

## Abstract

Ferredoxin:NADP^+^ oxidoreductase from *Plasmodium falciparum* (*Pf*FNR) catalyzes the NADPH-dependent reduction of ferredoxin (*Pf*Fd), which provides redox equivalents for the biosynthesis of isoprenoids and fatty acids in the apicoplast. Like other flavin-dependent electrontransferases, *Pf*FNR is a potential source of free radicals of quinones and other redox cycling compounds. We report here a kinetic study of the reduction of quinones, nitroaromatic compounds and aromatic *N*-oxides by *Pf*FNR. We show that all these groups of compounds are reduced in a single-electron pathway, their reactivity increasing with the increase in their single-electron reduction midpoint potential (*E*^1^_7_). The reactivity of nitroaromatics is lower than that of quinones and aromatic *N*-oxides, which is in line with the differences in their electron self-exchange rate constants. Quinone reduction proceeds via a ping-pong mechanism. During the reoxidation of reduced FAD by quinones, the oxidation of FADH^.^ to FAD is the possible rate-limiting step. The calculated electron transfer distances in the reaction of *Pf*FNR with various electron acceptors are similar to those of *Anabaena* FNR, thus demonstrating their similar “intrinsic” reactivity. Ferredoxin stimulated quinone- and nitro-reductase reactions of *Pf*FNR, evidently providing an additional reduction pathway via reduced *Pf*Fd. Based on the available data, *Pf*FNR and possibly *Pf*Fd may play a central role in the reductive activation of quinones, nitroaromatics and aromatic *N*-oxides in *P. falciparum*, contributing to their antiplasmodial action.

## 1. Introduction

The emergence of a malarial parasite *Plasmodium falciparum* resistance to available drugs, e.g., chloroquine or artemisinin ([1] and references therein), results in both a demand for new antimalarial agents and a better understanding of their mechanisms of action. *P. falciparum* is particularly vulnerable to oxidative stress, which might be caused by its lack of the antioxidant enzymes catalase and glutathione peroxidase [2]. For this reason, redox cycling compounds such as quinones, aromatic nitrocompounds and aromatic *N*-oxides, which frequently exhibit antiplasmodial in vitro activity at micromolar or lower concentrations, were a subject of great interest for a number of years ([3,4,5,6,7,8] and references therein). However, only fragmental data are available on their reactions with *P. falciparum* redox enzymes [6,9,10,11].

It is commonly accepted that the single-electron reduction of quinones and other classes of prooxidant compounds is performed by flavin-dependent dehydrogenases-electrontransferases such as NADPH:cytochrome P-450 reductase (P-450R), ferredoxin:NADP^+^ oxidoreductase (FNR) or NO-synthase (NOS) ([12,13,14] and references therein). These enzymes, working in conjunction with physiological single-electron acceptors, transform a two-electron (hydride) transfer into a single-electron one by stabilizing the neutral (blue) semiquinone form of the flavin nucleotide as the reaction intermediates [15,16,17].

In *P. falciparum*, an FAD-containing ferredoxin:NADP^+^ oxidoreductase (*Pf*FNR, EC 1.18.1.2) is localized in a nonphotosynthetic plastid organelle called apicoplast [18,19], which performs the biosynthesis of isoprenoids and fatty acids and is essential for the parasite’s survival. *Pf*FNR supplies redox equivalents to the apicoplast redox system via a Fe_2_S_2_-protein ferredoxin (*Pf*Fd) [18]. *Pf*Fd is characterized by a standard redox potential (*E*^0^_7.5_) of -0.26 V and possesses about 50% amino acid sequence homology with plant ferredoxins [18]. *Pf*FNR is characterized by *E*^0^_7_ = −0.28 V [19]; it possesses low homology (20–30%) with plant FNRs, displaying unique large insertions and deletions [20]. The protein complex formation is attributed to the electrostatic interaction between the basic residues of *Pf*FNR and acidic residues of *Pf*Fd and is sensitive to ionic strength [18,19,21].

*Pf*FNR reduces quinones and nitroaromatic compounds in a single-electron way and, based on currently available data, may be considered as an important source of their radicals in *P. falciparum* [6,11]. In this work, we extended the studies of *Pf*FNR using a large number of nonphysiological electron acceptors with different structures, reduction potentials and electrostatic charges. Our results provide a general insight into their reduction mechanisms and highlight the specific features of *Pf*FNR relevant to these processes.

## 2. Results

### 2.1. Steady-State Kinetics and Substrate Specificity Studies of PfFNR

**Figure 1:**
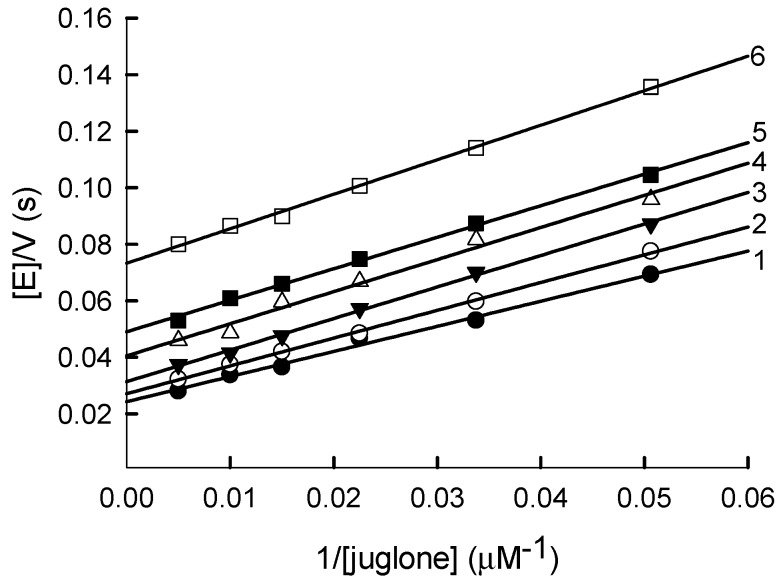
Steady-state kinetics of a reduction of juglone by NADPH catalyzed by *Pf*FNR. NADPH concentrations: 200 µM (1), 150 µM (2), 100 µM (3), 75 µM (4), 50 µM (5) and 25 µM (6).

In a previous study, juglone (5-hydroxy-1,4-naphthoquinone) was identified as one of the most active nonphysiological electron acceptors of *Pf*FNR [6]. In this work a series of parallel lines was obtained in double-reciprocal plots at varied concentrations of juglone and fixed concentrations of NADPH (Figure 1). This indicates that the quinone-reductase reaction catalyzed by *Pf*FNR follows a “ping-pong” mechanism. As deduced from Equation (A1) (Appendix A), the *k*_cat_ value for the juglone reduction at an infinite NADPH concentration is equal to 63.2 ± 4.1 s^−1^, and the values of the bimolecular rate constants (or catalytic efficiency constants, *k*_cat_/*K*_m_) for NADPH and juglone are equal to 6.0 ± 0.4 × 10^5^ M^−1^s^−1^ and 1.1 ± 0.1 × 10^6^ M^−1^s^−1^, respectively.

In order to assess the substrate specificity of *Pf*FNR, we examined the reduction of three series of electron acceptors, namely quinones (Q), nitroaromatic compounds (ArNO_2_) and aromatic *N*-oxides (ArN→O), whose single-electron reduction midpoint potentials (*E*^1^_7_) vary from 0.01 V to −0.575 V. In addition, several single-electron acceptors, ferricyanide, Fe(EDTA)^−^ and benzylviologen have been studied. The apparent reduction maximal rate constants, *k*_cat(app)_, of electron acceptors at 100 µM NADPH, and their respective *k*_cat_/*K*_m_, are given in Table 1. The *k*_cat_ values for a number of less-active oxidants were not determined because of a nearly linear dependence of the reaction rate on their concentrations.

**Figure 2:**
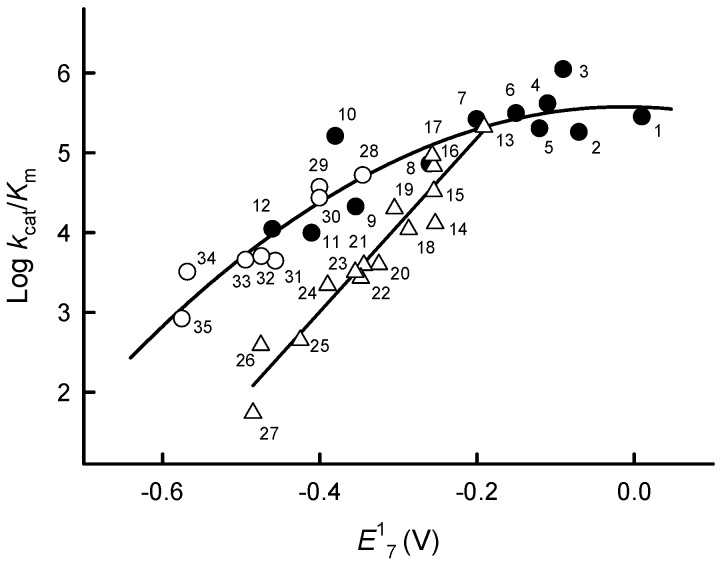
Dependence of the reactivity of quinones, nitroaromatic compounds and aromatic *N*-oxides on their single-electron reduction midpoint potentials. Relationship between the log *k*_cat_/*K*_m_ of quinones (solid circles), nitroaromatics (blank triangles) and *N*-oxides (blank circles) and their single-electron reduction midpoint potentials at pH 7.0 (*E*^1^_7_). Numbers and reduction potentials of compounds are given in Table 1.

**Figure 3:**
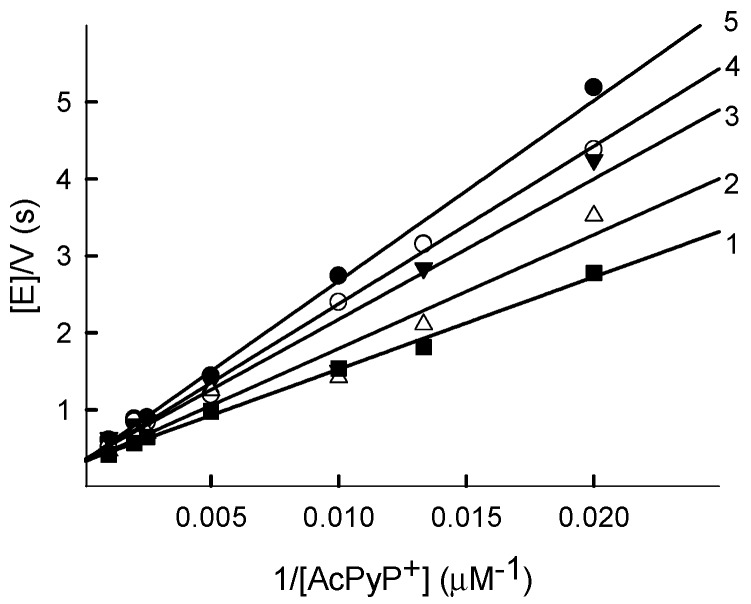
Steady-state kinetics of the reduction of 3-acetylpyridineadenine dinucleotide phosphate by *Pf*FNR. NADPH concentrations: 200 µM (1), 150 µM (2), 100 µM (3), 50 µM (4) and 25 µM (5).

The log *k*_cat_/*K*_m_ of ArNO_2_ exhibits a linear dependence on their *E*^1^_7_ (Table 1 and Figure 2). In general, the log *k*_cat_/*K*_m_ values of quinones and aromatic *N*-oxides are higher than those of nitroaromatics and are characterized by a parabolic dependence on their *E*^1^_7_ values (Figure 3). It is important to note that the reactivity of the single-electron acceptor benzylviologen matches the reactivity of quinones (Figure 2).

Previously, we found that *Pf*FNR reduces quinones and nitroaromatic compounds in a single-electron way [6,11]. Here, we found that the *Pf*FNR-catalyzed oxidation of NADPH by 100–200 µM tirapazamine was accompanied by O_2_ consumption at a rate close to that of NADPH oxidation. The addition of 50-µM cytochrome *c* to the reaction mixture resulted in its reduction at a rate representing 180–190% of that of NADPH oxidation. Superoxide dismutase (100 U/mL) inhibited the reduction of cytochrome *c* by 40–65%. This shows that the single-electron flux in the *Pf*FNR-catalyzed reduction of ArN→O is equal to 90–95% and that cytochrome *c* is reduced by their radicals, which are under a steady state with the O_2_/O_2_^−.^ couple.

Pyridine nucleotide analogues of NAD(P)^+^ are frequently used in the analysis of mechanisms of NAD(P)H-oxidizing flavoenzymes. The *k*_cat_ of the transhydrogenase reaction of *Pf*FNR, the formation of reduced 3-acetylpyridineadenine dinucleotide phosphate (AcPyPH) from AcPyP^+^ at the expense of NADPH, did not depend on the NADPH concentration, in the 25–200 µM range, and was equal to 3.2 ± 0.4 s^−1^ (Figure 3). However, in this case, the *k*_cat_/*K*_m_ for the oxidant decreased with the increase in the NADPH concentration (Figure 3). This shows that NADPH acts as a competitive inhibitor to AcPyP^+^, occupying the pyridine nucleotide binding site of the reduced enzyme form. As deduced from Equation (A2) (Appendix A), the *K*_is_ of NADPH, describing the effect of NADPH on the slopes in the Lineweaver–Burk plots, is equal to 140 ± 20 µM, and the *k*_cat_/*K*_m_ for AcPyP^+^ at (NADPH) = 0 is equal to 9.3 ± 0.8 × 10^3^ M^−1^s^−1^.

The affinity of *Pf*FNR for its physiological oxidant, *Pf*Fd, decreases with the ionic strength of the medium, due to the electrostatic character of their interaction [18,21]. In order to assess the role of electrostatic interactions in the reactions of *Pf*FNR with nonphysiological oxidants, we examined the effects of ionic strength on their reduction rates. It was reported that the NADPH-ferricyanide reductase reaction of *Pf*FNR was inhibited by high concentrations of ferricyanide, which acted as a competitive inhibitor with respect to NADPH (*K*_i_ = 230 µM) [20]. However, when the phosphate buffer was used instead of 0.1-M Tris-HCl [20], the substrate inhibition by ferricyanide was absent. This enabled us to perform a more thorough analysis of its reduction kinetics.

**Figure 4:**
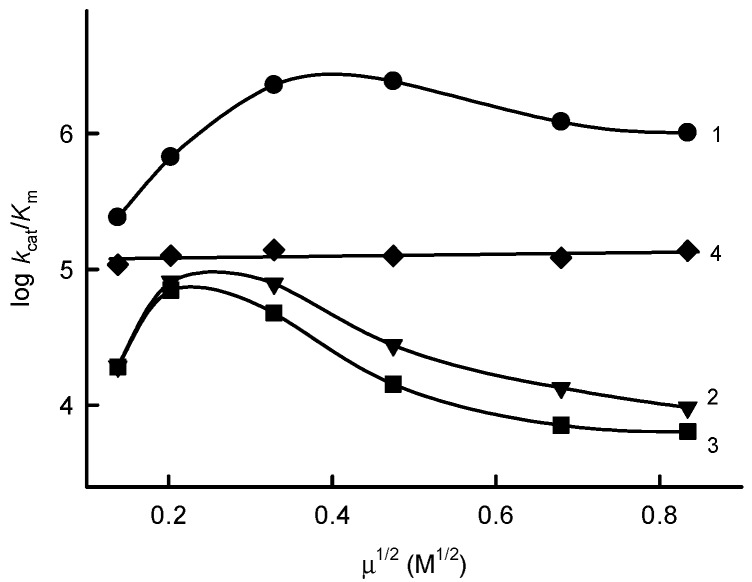
Effects of the ionic strength on the reactivity of PfFNR towards the electron acceptors. The dependence of log *k*_cat_/*K*_m_ for ferricyanide (1), Fe(EDTA)^−^ (2), benzylviologen (3) and tetramethyl-1,4-benzoquinone (4) on the ionic strength of the phosphate buffer at pH 7.0 is shown.

The data of Figure 4 show a bell-shape dependence of log *k*_cat_/*K*_m_ for ferricyanide, Fe(EDTA)^−^ and benzylviologen on the ionic strength of the solution, irrespective of the opposite electrostatic charge of the latter oxidant. In contrast, the *k*_cat_/*K*_m_ for the uncharged electron-acceptor tetramethyl-1,4-benzoquinone did not depend on the ionic strength (Figure 4).

### 2.2. Kinetics of PfFNR Oxidation under Multiple Turnover Conditions

**Figure 5:**
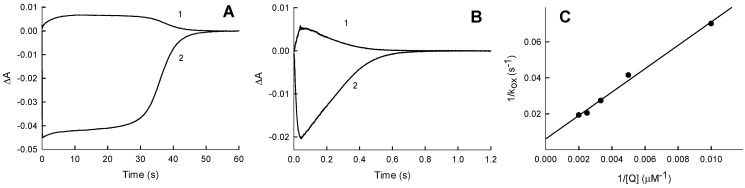
Turnover of PfFNR in the presence of NADPH and oxidants. (**A**,**B**) The kinetics of the absorbance changes at 600 nm (1) and 460 nm (2) during the reduction of Pf FNR (6.0 µM) by 50-µM NADPH and its subsequent reoxidation by oxygen (**A**) or 250-µM tetramethyl-1,4-benzoquinone (**B**) (concentrations after mixing). (**C**) The dependence of an apparent first-order reoxidation rate constant on the concentration of tetramethyl-1,4-benzoquinone.

In order to get insight into the enzyme reoxidation mechanism, we investigated the spectral changes of *Pf*FNR-bound FAD during its multiple turnover under aerobic conditions in the presence of NADPH and tetramethyl-1,4-benzoquinone. This electron acceptor does not absorb light at ≥460 nm, and its semiquinone form is rapidly reoxidized by oxygen [26]. In control experiments performed in the absence of quinone, the initial fast phase of FAD reduction by NADPH monitored at 460 nm is followed by its slower reoxidation by oxygen (Figure 5A). Importantly, this is accompanied by a transient increase in absorbance at 600 nm at the same time scale (Figure 5A). The addition of quinone accelerates the reoxidation of FADH^−^ and the decay of the 600-nm absorbing species by about two orders of magnitude (Figure 5B). This shows that, under our experimental conditions, O_2_ plays a negligible role in the kinetics of enzyme reoxidation. This process is also accompanied by a transient increase in absorbance at 600 nm. The kinetics of reoxidation were analyzed by the method of chance [27] using Equation (1), where *k*_ox_ is the apparent first-order rate constant of enzyme reoxidation, (NADPH)_0_ is the initial NADPH concentration, (E_red_)_max_ is the maximal concentration of the reduced enzyme formed during the turnover and t_1/2(off)_ is the time interval between the formation of the half-maximal amount of E_red_ and its decay to the half-maximal value:

(1) $$k\mathrm{ox}=\frac{{\left[\mathrm{NADPH}\right]}_{0}}{{\left[E_{\mathrm{red}}\right]}_{\max}\cdot t_{1/2\left(\mathrm{off}\right)}}\;$$

It was assumed that complete FAD reduction corresponds to the maximal ∆A_460_ after the enzyme mixing with NADPH in the absence of quinone (Figure 5A). This 460-nm absorbance change was close to that expected using the value of ∆ε_460_ = 7.8 mM^−1^cm^−1^ for the absorbance difference between the oxidized and two-electron reduced *Pf*FNR [19,28,29]. For the reoxidation of *Pf*FNR with oxygen (Figure 5A), we obtained a *k*_ox_ = 0.18 ± 0.02 s^−1^, which was close to the enzyme NADPH oxidase activity under a steady state. The dependence of *k*_ox_ on the tetramethyl-1,4-benzoquinone concentration (Figure 5C) gives an apparent bimolecular rate constant of 1.34 ± 0.37 × 10^5^ M^−1^s^−1^, which is comparable with the steady-state *k*_cat_/*K*_m_ for this oxidant (Table 1) and *k*_ox(max)_ = 155 ± 32 s^−1^. However, the later value may lack sufficient accuracy, because it was impossible to obtain a saturating concentration (sufficiently high *k*_ox_ values) due to the limited solubility of the oxidant.

### 2.3. NADP^+^ Inhibition Studies

**Figure 6:**
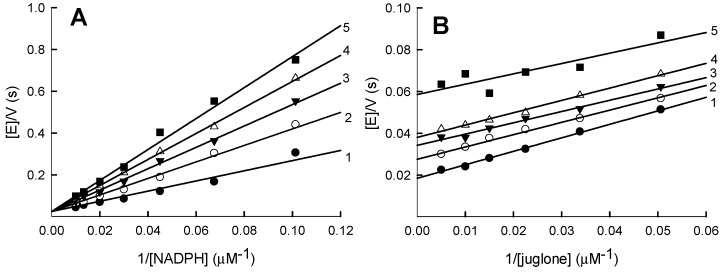
Inhibition of the juglone reductase reaction of *Pf*FNR by NADP^+^. (**A**) Inhibition at varied NADPH concentrations in the presence of 100-µM juglone, and (**B**) inhibition at varied juglone concentrations in the presence of 100-µM NADPH, NADP^+^ concentrations: 0 mM (1), 1.0 mM (2), 2.0 mM (3), 3.0 mM (4) and 5.0 mM (5).

At a fixed juglone concentration, the reaction product NADP^+^ acted as a competitive inhibitor towards NADH (Figure 6A) with *K*_is_ = 1.42 ± 0.13 mM, as deduced from Equation (A2) (Appendix A). In turn, at a fixed concentration of 50 µM NADH, NADP^+^ acted as an uncompetitive inhibitor towards juglone (Figure 6B) with *K*_ii_ = 1.83 ± 0.19 mM, as deduced from Equation (A3) (Appendix A), describing the effects of NADP^+^ on the intercepts in the Lineweaver–Burk plots. As compared with the previous data obtained in 0.05-M Hepes, the use of 0.1-M phosphate decreased the *k*_cat_/*K*_m_ for NADPH and increased the *K*_is_ of NADP^+^ almost by one order of magnitude [28,29].

### 2.4. Stimulation of Quinone- and Nitroreductase Activity of PfFNR by Ferredoxin

**Figure 7:**
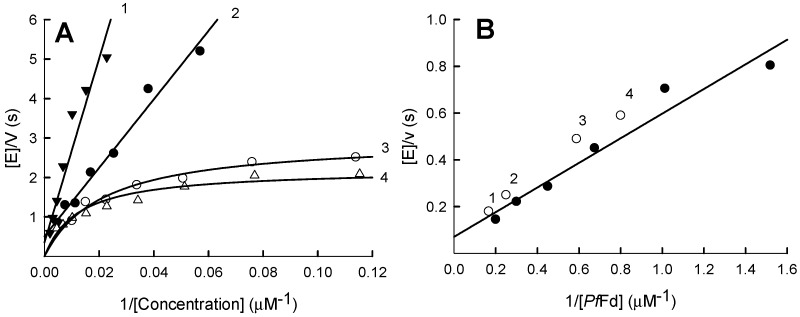
Stimulation of quinone- and nitroreductase reactions of *Pf*FNR by ferredoxin. (**A**) The dependence of the *Pf*FNR-catalyzed NADPH oxidation rate on the concentration of *p*-nitroacetophenone (1,4) or 2-hydroxy-1,4-naphthoquinone (2,3) in the absence of *Pf*Fd (1,2), and in the presence of 1.7-µM (3) or 4.0-µM (4) *Pf*Fd; concentration of NADPH is 100 µM. (**B**) The dependence of the cytochrome *c* reduction rate by *Pf*FNR on the concentration of *Pf*Fd (solid circles), concentration of NADPH, 100 µM and concentration of cytochrome *c,* 50 µM. Blank circles show the doubled maximal rates of the ”slower” phase of NADPH oxidation in the presence of tetramethyl-1,4-benzoquinone (1), *p*-nitroacetophenone (2,4) and 2-hydroxy-1,4-naphthoquinone (3) at corresponding concentrations of *Pf*Fd. The maximal rates were obtained by the fitting of kinetic data of the “slower” phase (5–6 lower concentrations of oxidant) to the parabolic expression.

The complex of *Pf*FNR with *Pf*Fd is characterized by micromolar *K*_d_ values [19,21]. Therefore, it is important to characterize the reduction of nonphysiological electron acceptors in the presence of both redox proteins. *Pf*Fd stimulated the reduction of quinones and nitroaromatics by *Pf*FNR, concomitantly causing a biphasicity of the corresponding Lineweaver-Burk plots (Figure 7A). The maximal rates of their “slower” phase (low concentrations and higher *k*_cat_/*K*_m_ of the oxidant) were close to the rates of cytochrome *c* reduction at corresponding *Pf*Fd concentrations (Figure 7B), which, in turn, are equal to the rate of *Pf*Fd reduction by *Pf*FNR. In 0.1-M K-phosphate, pH 7.0, the maximal rate of this reaction at saturating the *Pf*Fd concentration is 16 ± 2.5 s^−1^, on the one-electron base, which is close to the previously determined values, 13–15 s^−1^ [19]. The *K*_m(app)_ for *Pf*Fd is 5.2 ± 1.3 µM, a value significantly higher than that previously reported [19], which may be attributed to the higher ionic strength of the medium.

## 3. Discussion

These results provide a general insight into the mechanism of reduction of several groups of nonphysiological oxidants by *Pf*FNR and complement the data on the mechanism of its interaction with the physiological electron-acceptor *Pf*Fd [18,19]. First, let us consider the possible rate-limiting step of the reaction.

Since *Pf*FNR follows a ping-pong mechanism (Figure 1), its reductive and oxidative half-reactions can proceed independently, with the *k*_cat_ expressed as 1/*k*_cat_ = 1/*k*_red(max)_ + 1/*k*_ox(max)_, where *k*_red(max)_ and *k*_ox(max)_ are the maximal rates of the reductive and oxidative half-reactions, respectively. Our data show that, in the reduction of tetramethyl-1,4-benzoquinone, the overall catalytic process should be partly limited by both oxidative and reductive half-reactions, because the maximal rate of *Pf*FNR reduction by NADPH at pH 7.0 is 125–148 s^−1^ [19,29], and a comparable value, *k*_ox(max)_ = 155 ± 32 s^−1^, was obtained for tetramethyl-1,4-benzoquinone under multiple turnover conditions (Figure 5C). On the other hand, the rate of the enzyme reduction with a fixed concentration of NADPH should be the same, irrespectively of the oxidant used, and the observed *k*_cat(app)_ differences (Table 1) should be attributed to different maximal rates of reoxidation. Thus, for oxidants 1–4, 7, 10 and 13, whose *k*_cat(app)_ values are close to that of tetramethyl-1,4-benzoquinone (Table 1), the overall catalytic process should be partly limited by both oxidative and reductive reactions, as well. The lower values of *k*_cat(app)_ for other compounds (Table 1) imply that, in these cases, the process is limited by the oxidative half-reaction. The nature of these differences will be the object of our future studies.

Since reduced PfFNR is reoxidized in a single-electron fashion, the oxidative half-reaction must proceed through the two steps of FADH^−^ → FADH^.^ and FADH^.^ →FAD. There are several arguments supporting FAD semiquinone oxidation as a possible rate-limiting step of the overall process: (a) The 600-nm absorbing species is formed during the reoxidation of reduced *Pf*FNR by quinones, which may point to a transient FADH^.^ accumulation [13,30] (Figure 5B); (b) NADPH acts as a competitive inhibitor with respect to the oxidant in the reduction of AcPyP^+^ by *Pf*FNR (Figure 3). In contrast, NADPH does not inhibit quinone reduction (Figure 1). This shows that quinones and AcPyP^+^ oxidize different redox forms of *Pf*FNR, which possess different affinities for NADPH. Since AcPyP^+^ is an obligatory two-electron (hydride) acceptor, it can be reduced only by a two-electron reduced FAD. This argues against its involvement as a rate-limiting step in quinone reduction, and (c) NADP^+^ acts as a competitive inhibitor with respect to NADPH (Figure 6A) and as an uncompetitive inhibitor with respect to quinones (Figure 6B). This may be attributed to a specific case of a ping-pong mechanism, where NADP^+^ binds relatively tightly to the oxidized enzyme form but binds weakly or not at all to its reduced state [31]. Since NADP^+^ binds tightly to the two-electron reduced form of *Pf*FNR [28,29], this also argues against its involvement as a rate-limiting step in quinone reduction. Thus, *Pf*FNR may have properties in common with FNRs from *Anabaena* PCC7118. and from spinach, where the oxidation of FADH^.^ is the rate-limiting step of reactions with various nonphysiological electron acceptors [13,30,32,33,34].

Moreover, our study discloses some specific properties of *Pf*FNR relevant to the reduction of nonphysiological redox agents. The experiments at varied ionic strengths may characterize the surface region of *Pf*FNR that interacts with charged oxidants. In this regard, the observed bell-shape dependences of the reactivity of oppositely charged oxidants on the ionic strength of the solution (Figure 4) were unexpected. Similar, although less pronounced, dependences were observed in the reactions of FNR from *Anabaena* PCC7118 [13]. A possible explanation of this phenomenon is that the oxidants may interact with both the negatively charged Glu-314 (Glu-301 in *Anabaena* PCC7118 FNR [18]) and positively charged Lys-287 (Arg-274 in *Anabaena* PCC7118 FNR), the latter participating in the binding of *Pf*Fd, which are located close to the dimethylbenzene part of the isoalloxazine ring [20,21]. This is in line with the finding that ferricyanide competes with the nicotinamide mononucleotide part of NADP^+^ for binding close to the FAD isoalloxazine ring [20]. Other positively charged residues participating in the binding of *Pf*Fd, such as Arg-98, Arg-290 and Lys-308, are too distant from the isoalloxazine ring [21] and may not be likely to interact with low molecular weight oxidants.

Our data point to an absence of a strict substrate specificity in the reduction of quinones, ArNO_2_, and ArN→O by *Pf*FNR, with the exception of an increase in their log *k*_cat_/*K*_m_ with an increase in *E*^1^_7_ (Figure 3). The latter feature points to an “outer-sphere” electron transfer mechanism of their reduction, which is established for FNR from *Anabaena* PCC7119, P-450R and NOS [12,13,14]. According to this mechanism, the bimolecular rate constant of the electron transfer between the reactants (*k*_12_) is expressed as:

*k*_12_ = (*k*_11_ × *k*_22_ × *K* × *f*)^1/2^, (2)

where *k*_11_ and *k*_22_ are the electron self-exchange rate constants of the reactants, *K* is the equilibrium constant of the reaction (log *K* = Δ*E*^1^/0.059 V) and *f* is expressed as:

log *f* = (log *K*)^2^/4log (*k*_11_ × *k*_22_/*Z*^2^), (3)

where *Z* is the frequency factor, 10^11^ M^−1^s^−1^ [35]. According to Equations (2 and 3), in the reaction of the electron donor with a series of homologous electron acceptors (which display the same *k*_22_), log *k*_12_ will exhibit a parabolic (quadratic) dependence on ∆*E*^1^ with a slope 8.45 V^−1^ at ∆*E*^1^ = ±0.15 V. In particular, the lower reactivity of ArNO_2_ as compared with quinones and ArN→O, which possess similar *E*^1^_7_ values (Figure 3), is explained by their *k*_22_ = ~10^6^ M^−1^s^−1^ [36], which are much lower than those of quinones and ArN→O, ~10^8^ M^−1^s^−1^ [36,37].

According to the model of Mauk et al. [38], at an infinite ionic strength, where electrostatic interactions are absent, the *k*_11_ value of metalloproteins for the reactions with the inorganic complexes is related to the distance of the electron transfer, *R*_p_:

*R*_p_ (Å) = 6.3 − 0.35 ln *k*_11_ (4)

We have applied this approach for the analysis of the single-electron oxidation of P-450R, FNR from *Anabaena* PCC7118 and NOS by quinones, nitroaromatics and inorganic complexes [12,13,14]. Their reactions with Q and ArNO_2_ are characterized by *R*_p_ ranging from 3.4 to 5.0 Å, whereas, for the reactions with hydrophilic ferricyanide and Fe(EDTA)^−^, which are incapable of entering the protein globule, the *R*_p_ values are much higher (Table 2). However, it is possible that this procedure gives slightly overestimated distances in the case of flavoproteins, since the dimethylbenzene part of the flavin isoalloxazine ring in P-450R, FNR and NOS is partly exposed to the solvent [39,40,41]. Thus, these values may be useful only for an approximate assessment of the “intrinsic” flavoenzyme reactivity. The estimation of *k*_11_ and *R*_p_ for the reactions of *Pf*FNR is complicated by the unknown *E*^1^_7_ value for the FAD/FADH^.^ couple. Based on the available data [19], the FAD semiquinone state in *Pf*FNR is not stabilized. Thus, the values of *k*_11_ were calculated tentatively assuming 15% and 5% FAD^.^ formations at the equilibrium (Appendix B). The resulting values of *R*_p_ for *Pf*FNR are given in Table 2.

Typically, they are larger than those of P-450R but close to those of FNR from *Anabaena* PCC7118. This shows that low molecular weight oxidants may access the FAD isoalloxazine ring of both representatives of FNR with similar ease, in spite of some differences in their surroundings [20,21,41].

Finally, the stimulation of the nonphysiological acceptor reductase reactions of *Pf*FNR by *Pf*Fd (Figure 7A,B) shows that *Pf*Fd provides an alternative pathway for their reduction via reduced *Pf*Fd. Since the redox potentials of both proteins are similar [18,19], this may be most easily explained by a better accessibility of the active center of *Pf*Fd. This phenomenon has been previously observed in reactions of bovine adrenodoxin reductase and adrenodoxin and *Anabaena* PCC7118 FNR and Fd [13,42]; thus, it may be a general feature of this group of redox proteins.

In conclusion, our comprehensive study characterized the mechanism of reactions of *Pf*FNR with redox-cycling xenobiotics, which may be instrumental in the further development of redox-active antiplasmodial agents [3,4,5,6,7,8]. In terms of *k*_cat_/*K*_m_ for the reduction of quinones and nitroaromatics (Table 1 and Figure 2), the reactivity of *Pf*FNR is considerably higher as compared to other *P. falciparum* flavoenzymes, glutathione reductase, thioredoxin reductase and type 2 NADH dehydrogenase [9,10]. To the best of our knowledge, the reactions of the above groups of xenobiotics with other electrontransferases of the *P. falciparum* mitochondrial respiratory chain, namely dihydroorotate dehydrogenase, succinate dehydrogenase and malate:quinone oxidoreductase, have not been studied so far. Thus, based on the available data, *Pf*FNR and possibly *Pf*Fd may play a central role in the reductive activation of pro-oxidant xenobiotics relevant for malaria chemotherapy. This also points to a versatility of the properties of *Pf*FNR, which may be relevant for the design of new antiplasmodial agents, because another intriguing approach to this task is the development of compounds that may bind at the *Pf*FNR-*Pf*Fd interface and inhibit the physiological reduction of *Pf*Fd [43,44]. Thus, an attractive possibility could be the derivatization of these ligands, chalcones or alkaloids [43,44] by redox-active quinone, ArNO_2_ or ArN→O moieties. These hybrid molecules may combine two mechanisms of antiplasmodial action: the inhibition of the electron supply to the apicoplast redox system and redox cycling.

## 4. Materials and Methods

### 4.1. Enzymes and Reagents

**Figure 8:**
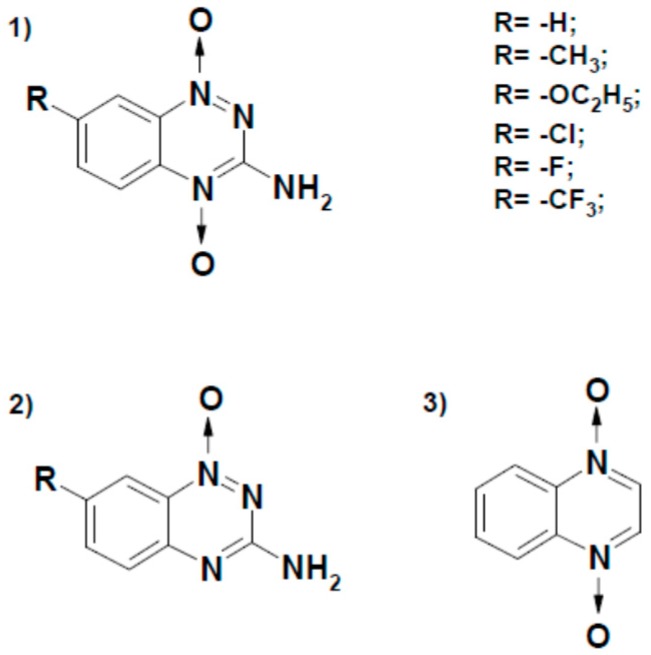
Structural formulae of aromatic *N*-oxides used in this work. Derivatives of 3-amino-1,2,4-benzotriazine-1,4-dioxide (tirapazamine) (1), 3-amino-1,2,4-benzotriazine 1-oxide (2) and quinoxaline-1,4-dioxide. Formulae were drawn using ISIS/Draw (v. 2002, MDL Information Systems, San Leandro, CA, USA).

Recombinant *P. falciparum* ferredoxin:NADP^+^ oxidoreductase and ferredoxin were prepared as previously described [19], and their concentrations were determined spectrophotometrically according to ***ε***_454_ = 10.0 mM^−1^ cm^−1^ and ε_424_ = 9.68 mM^−1^ cm^−1^, respectively. Compounds 2,4,6-trinitrotoluene (TNT) and 2,4,6-trinitrophenyl-*N*-methylnitramine (tetryl) were synthesized as described [45]. The 5-Nitrothiophene-2-carbonic acid morpholide was synthesized as described [25]. The 7-substituted tirapazamines, quinoxaline 1,4-dioxide and 1-oxide of tirapazamine (Figure 8) were synthesized as described [23,46,47].

All compounds were characterized by determining their melting point, as well as their ^1^H-NMR, UV and IR spectra. The purity of the compounds, determined using a high-performance liquid chromatography system equipped with a mass spectrometer (LCMS-2020, Shimadzu, Kyoto, Japan), was >98%. Cytochrome ***c***, NADPH, superoxide dismutase and other compounds were obtained from Sigma-Aldrich (St. Louis, MO, USA) and used as received.

### 4.2. Steady-State Kinetic Studies

All kinetic experiments were carried out spectrophotometrically using a PerkinElmer Lambda 25 UV–VIS spectrophotometer (PerkinElmer, Waltham, MA, USA) in 0.1-M K-phosphate buffer (pH 7.0) containing 1-mM EDTA at 25 °C. The steady-state parameters of the reactions, the catalytic constants (*k*_cat(app.)_) and the bimolecular rate constants (or catalytic efficiency constants, *k*_cat_/*K*_m_) of the oxidants at fixed concentrations of NADPH were obtained by fitting the kinetic data to the parabolic expression using SigmaPlot 2000 (v. 11.0, SPSS Inc., Chicago, IL, USA). They correspond to the reciprocal intercepts and slopes of Lineweaver-Burk plots, (E)/V vs. 1/(oxidant) respectively, where V is the reaction rate, and (E) is the enzyme concentration. *k*_cat_ represents the number of molecules of NADPH oxidized by a single active center of the enzyme per second. In the case of two-substrate reactions or inhibitions, the data were fitted to Equations (A1–3). The rates of *Pf*FNR-catalyzed NADPH oxidation in the presence of quinones, nitroaromatic compounds or tirapazamine derivatives were determined using the value Δ***ε***_340_ = 6.2 mM^−1^ cm^−1^. The rates were corrected for the intrinsic NADPH-oxidase activity of the enzyme, determined as 0.12 s^−1^. In separate experiments, in which 50-µM cytochrome ***c*** was included in the reaction mixture, its tirapazamine-mediated reduction was measured using the value Δ***ε***_550_ = 20 mM^−1^ cm^−1^. Ferricyanide reduction rate was determined using the value ∆ε_420_ = 1.03 mM^−1^cm^−1^. The reduction rate of AcPyP^+^ was determined using the value ∆ε_363_ = 5.6 mM^−1^cm^−1^ [48]. The rates of oxygen consumption during the reactions were monitored under identical conditions using a Clark electrode (Rank Brothers Ltd., Bottisham, UK).

### 4.3. Presteady-State Kinetic Studies

Enzyme rapid kinetic studies were performed using a SX20 stopped-flow spectrophotometer (Applied Photophysics, Leatherhead, UK) under aerobic conditions. The enzyme reduction by NADPH and its reoxidation was monitored at 460 and 600 nm, respectively. During turnover studies, the enzyme in the first syringe (6.0–7.0 µM after mixing) was mixed with the contents of the second syringe (50-µM NADPH and 100–500-µM tetramethyl-1,4-benzoquinone after mixing).

## Figures and Tables

**Table 1:** Steady-state rate constants of the reduction of nonphysiological electron acceptors by NADPH catalyzed by *Pf*FNR. (NADPH) = 100 µM, 0.1-M K-phosphate + 1.0-mM EDTA, pH 7.0, 25 °C.

No.	Compound	*E*^1^_7_ (V) [22,23,24,25]	*k*_cat(app)_ (s^−1^)	*k*_cat_/*K*_m_ (M^−1^s^−1^)
Quinones				
1	2-Methyl-1,4-benzoquinone	0.01	32.4 ± 4.1	2.8 ± 0.2 × 10^5^
2	2,5-Dimethyl-1,4-benzoquinone	−0.07	26.0 ± 2.3	1.8 ± 0.2 × 10^5^
3	5-Hydroxy-1,4-naphthoquinone	−0.09	35.7 ± 5.1	1.1 ± 0.1 × 10^6^
4	5,8-Dihydroxy-1,4-naphthoquinone	−0.11	25.0 ± 3.8	4.1 ± 0.5 × 10^5^
5	9,10-Phenanthrene quinone	−0.12	20.3 ± 3.3	2.0 ± 0.3 × 10^5^
6	1,4-Naphthoquinone	−0.15	16.9 ± 2.1	3.1 ± 0.3 × 10^5^
7	2-Methyl-1,4-naphthoquinone	−0.20	26.5 ± 3.2	2.6 ± 0.3 × 10^5^
8	Tetramethyl-1,4-benzoquinone	−0.26	33.1 ± 4.3	7.2 ± 0.8 × 10^4^
9	Benzylviologen	−0.354	4.8 ± 0.6	2.1 ± 0.1 × 10^4^
10	9,10-Anthraquinone-2-sulphonate	−0.38	27.0 ± 3.2	1.6 ± 0.2 × 10^5^
11	2-Hydroxy-1,4-naphthoquinone	−0.41	2.6 ± 0.3	9.8 ± 0.2 × 10^3^
12	2-Methyl-3-hydroxy-1,4-naphthoquinone	−0.46	14.0 ± 1.2	1.1 ± 0.1 × 10^4^
Nitroaromatic Compounds				
13	Tetryl	−0.191	40.0 ± 5.1	2.1 ± 0.4 × 10^5^
14	2,4,6-Trinitrotoluene ^a^	−0.253		1.3 ± 0.3 × 10^4^
15	Nifuroxime ^a^	−0.255		3.3 ± 0.2 × 10^4^
16	Nitrofurantoin ^a^	−0.255		6.8 ± 0.5 × 10^4^
17	1,4-Dinitrobenzene	−0.257		9.1 ± 0.8 × 10^4^
18	1,2-Dinitrobenzene ^a^	−0.287		1.1 ± 0.2 × 10^4^
19	5-Nitrothiophene-2-carbonic acid morpholide	−0.305		2.0 ± 0.2 × 10^4^
20	4-Nitrobenzaldehyde ^a^	−0.325		4.0 ± 0.3 × 10^3^
21	3,5-Dinitrobenzoic acid ^a^	−0.344		3.9 ± 0.5 × 10^3^
22	1,3-Dinitrobenzene ^a^	−0.348		2.7 ± 0.2 × 10^3^
23	4-Nitroacetophenone ^a^	−0.355		3.2 ± 0.4 × 10^3^
24	2-Nitrothiophene	−0.390		2.2 ± 0.2 × 10^3^
25	4-Nitrobenzoic acid ^a^	−0.425		4.5 ± 0.4 × 10^2^
26	4-Nitrobenzyl alcohol	−0.475		3.9 ± 0.2 × 10^2^
27	Nitrobenzene ^a^	−0.485		5.5 ± 0.6 × 10^1^
Aromatic *N*-Oxides				
28	7-CF_3_-tirapazamine	−0.345	11.5 ± 2.0	5.2 ± 0.4 × 10^4^
29	7-Cl-tirapazamine	−0.400	14.8 ± 1.3	3.7 ± 0.4 × 10^4^
30	7-F-tirapazamine	−0.400		2.7 ± 0.2 × 10^4^
31	3-Amino-1,2,4-benzotriazine-1,4-dioxide (tirapazamine)	−0.456		4.4 ± 0.5 × 10^3^
32	7-CH_3_-tirapazamine	−0.474		5.0 ± 0.6 × 10^3^
33	7-C_2_H_5_O-tirapazamine	−0.494		4.5 ± 0.5 × 10^3^
34	3-Amino-1,2,4-benzotriazine-1-oxide	−0.568		3.2 ± 0.2 × 10^3^
35	Quinoxaline-1,4-dioxide	−0.575		8.2 ± 0.9 × 10^2^
Inorganic Complexes				
36	Ferricyanide ^b^	0.41	47.9 ± 4.0	3.0 ± 0.4 × 10^6^
37	Fe (EDTA)^−^	0.12		4.3 ± 0.2 × 10^4^

^a^ Taken from Reference [11]. ^b^ Calculated on a single-electron base. Catalytic efficiency constants, *k*_cat_/*K*_m_; reduction maximal rate constants, *k*_cat(app)_ and single-electron reduction midpoint potentials, *E*^1^_7_.

**Table 2:** Distances of the electron transfer (*R*_p_) in reactions of flavin-dependent electrontransferases with nonphysiological electron acceptors, calculated according to Equation (4).

Flavoprotein	Reaction	*R*_p_ (Å)			
		Q	ArNO_2_	Fe (CN)^3−^_6_	Fe (EDTA)^−^
P-450R, rat [12]	FMNH^−^ − e^−^ → FMNH^.^, *E*^1^_7_ = −0.270 V	3.4	4.2	8.1	7.3
*n*-NOS, rat [14]	FMNH^−^ − e^−^ → FMNH^.^, *E*^1^_7_ = −0.274 V	4.7	3.9	-	-
FNR, *Anabaena* PCC7118 ^a^	FADH^.^ − e^−^ − H^+^ → FAD, *E*^1^_7_ = −0.280 V	5.0	4.4	9.2	10.4–11.4
*Pf*FNR, this work	FADH^.^ − e^−^ − H^+^ → FAD, *E*^1^_7_ = −0.308 V ^b^	4.8	4.9	9.5	9.1
	FADH^.^ − e^−^ − H^+^ → FAD, *E*^1^_7_ = −0.337 V ^c^	5.0	5.6	9.8	9.4

^b^ Calculated according to the data of Reference [13], using the value of *E*_7_ (FAD/FADH^.^) = −0.280 V [30]. ^b,c^ The values of *E*^1^_7_ are calculated assuming 15% and 5% FADH^.^ stabilizations at the equilibrium, respectively.

## Abbreviations

AcPyP^+^	3-Acetylpyridineadenine dinucleotide phosphate
ArNO_2_	aromatic nitrocompound
ArN→O	aromatic *N*-oxide
*E* ^1^_7_	single-electron reduction midpoint potential at pH 7.0
E^0^_7_	two-electron (standard) reduction midpoint potential at pH 7.0
Fd	ferredoxin
FNR	ferredoxin:NADP^+^ oxidoreductase
*k*_cat_	catalytic constant
*k*_cat_/*K*_m_	bimolecular rate constant
Q	quinone

## Appendix A

Kinetic parameters of steady-state reactions according to a ping-pong mechanism were calculated according to Equation (A1):

(A1) $$\frac{V}{\left[E\right]}=\frac{k_{cat\;}\left[S\right]\left[Q\right]}{K_{m\left(S\right)}\left[Q\right]+K_{m\;\left(Q\right)}\left[S\right]+\left[S\right]\left[Q\right]}$$

where S stands for NADPH, and Q stands for the electron acceptor. The competitive inhibition constant (*K*_is_) of NADP^+^ (I) vs. NADPH (S) was calculated according to Equation (A2):

(A2) $$\frac{V}{\left[E\right]}=\frac{k_{\mathrm{cat}}\;\left[S\right]}{K_{m\left(S\right)}\left(1+\frac{\left[I\right]}{K_{\mathrm{is}}}\right)+\left[S\right]}\;$$

For the transhydrogenase reaction, S stands for AcPyP^+^, and I stands for NADPH. The noncompetitive inhibition constant (*K*_ii_) of NADP^+^ vs. the electron acceptor was calculated according to Equation (A3):

(A3) $$\frac{V}{\left[E\right]}=\frac{k_{\mathrm{cat}}\;\left[Q\right]}{K_{m\left(Q\right)}+\left[Q\right]\left(1+\frac{\left[I\right]}{K_{\mathrm{ii}}}\right)}$$

## Appendix B

The *k*_22_ values of ferricyanide and Fe(EDTA)^−^at infinite ionic strengths are equal to 4.6 × 10^5^ M^−1^s^−1^ and 6.9 × 10^4^ M^−1^s^−1^, respectively [38]. At the ionic strength of the solution, 1.10 M, the *k*_12_ values of ferricyanide and Fe(EDTA)^−^ are equal to 1.0 × 10^6^ M^−1^s^−1^ and 1.0 × 10^4^ M^−1^s^−1^, respectively (Figure 4). Using E_7_(FAD/FADH^−^) = −0.28 V for *Pf*FNR [19], the calculations according to the Nernst equation give E_7_(FAD/FADH^.^) = −0.308 V (15% FADH^.^ stabilization) and E_7_(FAD/FADH^.^) = −0.337 V (5% FADH^.^ stabilization). The electron self-exchange rate constants of *Pf*FNR, *k*_11_, are calculated after the rearrangement of Equation (3) [49]:

log *k*_11_ = log *k*_12_ − log *k*_22_ − 0.5 log *K* + log *Z −* [(log *Z* − log *k*_12_)^2^ + log *K* (log *Z* − log *k*_12_)]^1/2^ (A4)

For the reactions of *Pf*FNR with ferricyanide, Equation (A4) gives *k*_11_ = 10^−4^ M^−1^s^−1^ (15% FADH^.^ stabilization) and *k*_11_ = 4.1 × 10^−5^ M^−1^s^−1^ (5% FADH^.^ stabilization). Similarly, for the reactions with Fe(EDTA), it yields *k*_11_ = 3.5 × 10^−4^ M^−1^s^−1^ (15% FADH^.^ stabilization) and *k*_11_ = 1.3 × 10^−4^ M^−1^s^−1^ (5% FADH^.^ stabilization). For the reactions of *Pf*FNR with Q and ArNO_2_, the approximate *k*_11_ values may be estimated from the data of Figure 3 at ∆*E*^1^_7_ = 0, where *k*_12_ = (*k*_11_ × *k*_22_)^1/2^. At a 15% FADH^.^ stabilization, i.e., at the *E*^1^_7_ of the oxidant being equal to −0.308 V, the *k*_11_ values are equal to 79 M^−1^s^−1^ (quinones) and to 63 M^−1^s^−1^ (nitroaromatics). At a 5% FADH^.^ stabilization, i.e., at the *E*^1^_7_ of the oxidant being equal to −0.337 V, the *k*_11_ values are equal to 40 M^−1^s^−1^ (quinones) and to 6.3 M^−1^s^−1^ (nitroaromatics).
